# Challenges and solutions to embed cancer survivorship research and innovation within the EU Cancer Mission

**DOI:** 10.1002/1878-0261.13022

**Published:** 2021-06-15

**Authors:** Mark Lawler, Francesco De Lorenzo, Pernilla Lagergren, Francesco S. Mennini, Saronas Narbutas, Grazia Scocca, Françoise Meunier, the European Academy of Cancer Sciences

**Affiliations:** ^1^ Patrick G Johnston Centre for Cancer Research Queen's University Belfast UK; ^2^ European Cancer Organisation Brussels Belgium; ^3^ European Cancer Patient Coalition Brussels Belgium; ^4^ Italian Federation of Cancer Patients Organisations Rome Italy; ^5^ Surgical Care Science Department of Molecular Medicine and Surgery Karolinska Institutet Karolinska University Hospital Stockholm Sweden; ^6^ Department of Surgery and Cancer Imperial College London UK; ^7^ EEHTA CEIS, DEF Department Faculty of Economics University “Tor Vergata” Rome Italy; ^8^ Institute of Leadership and Management in Health Kingston University London UK; ^9^ Lithuanian Cancer Patient Coalition Vilnius Lithuania; ^10^ Federation of European Academies of Medicine Brussels Belgium

**Keywords:** cancer survivorship, EU Cancer Mission, Europe's Beating Cancer Plan, living with and beyond cancer, survivorship research

## Abstract

We have reached a watershed moment in Europe in our efforts to ensure increased survival and better outcomes for cancer patients. The EU Cancer Mission and the European Beating Cancer Plan together provide an unrivalled opportunity to make significant inroads into a disease that kills over 1.7 million European citizens annually. Harnessing these twin pillars of cancer research and cancer control can be transformative for the European cancer community and in particular for the European cancer patient. However, from a research perspective, in order to fully realise these benefits, we need to ensure that all aspects of the cancer continuum are addressed. Previous research efforts have focussed more on the diagnosis and treatment of cancer, whereas cancer survivorship, to date, has been overlooked. Here, we aim to redress this balance, by identifying the key challenges in cancer survivorship research that need to be addressed and proposing a series of recommended solutions, which, if acted upon, would deliver significant benefits for the nearly 20 million cancer survivors in Europe. To achieve this, we propose the development of a clearly articulated and sustainably funded European Cancer Survivorship Research and Innovation Plan. Embedding this plan within the framework of the EU Cancer Mission would be transformative for cancer survivors and society.

## Graphical abstract

Previous research efforts have focussed more on the diagnosis and treatment of cancer, and less so on cancer survivorship. Here, we identify the key challenges in cancer survivorship research and propose the development of a clearly articulated and sustainably funded European Cancer Survivorship Research and Innovation Plan to deliver significant benefits for the nearly 20 million cancer survivors in Europe.

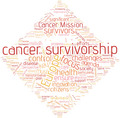

**Abbreviations**
CEE: Central and Eastern Europe
COVID‐19: severe acute respiratory syndrome coronavirus 2 (SARS‐CoV‐2)
EU: European Union
HRQoL: health‐related quality of life
SIOPE: European Society for Paediatric Oncology
US: United States

## 1 Introduction

Despite the undoubted progress that has been made in improving cancer control and increasing cancer survivorship over the last 50 years, the significant challenges that still persist across the cancer continuum have led to a renewed global focus on a disease that kills over 10 million citizens worldwide each year (https://gco.iarc.fr/). Critically, there has been a welcome realisation that research and innovation must be a key cornerstone of modern cancer control, with an undeniable body of evidence, indicating that those cancer patients who are treated in research‐active hospitals or centres achieve better outcomes than those who are not [1]. In the United States, the launch of the Cancer Moonshot and its continuing implementation has resonated with patients, clinicians and scientists, acting as a crucial driver in positioning research and innovation at the forefront of the fight against cancer [2]. In Europe, steady progress in cancer research and its translation into effective cancer care has led to improved outcomes for cancer patients [3], including childhood and young adult patients. However, the ageing population demographic, allied to genomic, environmental and lifestyle factors that will contribute to an increased incidence of cancer in the next 15–20 years, together with the challenges of treating the disease as it becomes more aggressive, mean that cancer and its control must be the critical focus for research and innovation implementation, health service delivery and health policy efforts over the coming years.

## 2 Ensuring cancer is at the top of the European health and research agenda

In Europe, the importance of ensuring that cancer is at the top of the health and research agenda has been recognised at the very highest political level. The President of the European Commission, Ursula Van Der Leyden, has emphasised that health is a clear priority within the agenda of the current European Parliament and has tasked the EU Commissioner for Health and Food Safety, Stella Kyriakides, to ensure that cancer is a disease of key focus in the European Commissioner's overarching strategy for Health (https://ec.europa.eu/health/non_communicable_diseases/events/ev_20200204_en). The recent launch of Europe's Beating Cancer Plan on the 3 February 2021 (https://ec.europa.eu/health/sites/health/files/non_communicable_diseases/docs/eu_cancer‐plan_en.pdf) underpins the commitment to cancer and represents a huge opportunity to deliver optimal care for cancer patients and enhanced support for cancer survivors across the European continent. From a research perspective, the EU Cancer Mission, one of only 5 Research Missions of the EU (https://ec.europa.eu/info/horizon‐europe/missions‐horizon‐europe_en), emphasises the need for a real step change in cancer research activity in Europe [4], supported through the upcoming Horizon Europe research funding programme. The Lancet Oncology European Cancer Groundshot Commission [5] also highlights the absolute primacy of cancer research and emphasises how deployment of data intelligence to identify the key research gaps and challenge that we face can illuminate the path to a vibrant European cancer research agenda, with a particular focus on Central and Eastern Europe (CEE). Additionally, the adverse impact of the COVID‐19 pandemic on cancer patients, cancer services and cancer research has been significant [6]; it is vital that the European Beating Cancer Plan and the EU Cancer Mission address both the short and longer term effects of the pandemic aggressively. In this regard, the recent launch by the European Cancer Organisation's Special Focussed Network on COVID‐19 and Cancer of a 7‐point plan to ‘Build Back Better’ (https://cancerworld.net/building‐back‐cancer‐services‐after‐covid‐19‐european‐cancer‐organisation‐plan/) can be both instructive and transformative.

## 3 Cancer in Europe: responding to the survivorship challenge

Although much progress has been made in delivering improved cancer control in Europe, there are still many challenges to be overcome, a number of which relate to the unequal nature of the cancer burden and its control in different parts of Europe, particularly in CEE. These inequalities [7] and disparities [8] that European citizens and cancer patients have been experiencing, allied to the increasing impact of the social determinants of health [9], mean that we must redouble our efforts to ensure that the following key areas are addressed from cancer control, cancer research and cancer policy perspectives:

i. that cancer prevention receives a higher priority from a public health, research and policy perspective;
ii. that citizens with suspicion of cancer are diagnosed at the earliest possible stage of their illness;
iii. that patients with cancer receive the optimal treatment and supportive care in a timely fashion, that is underpinned by the latest research and innovation;
iv. that cancer survivors are adequately supported in a holistic way to ensure that they experience the best possible quality of life, receive the appropriate rehabilitation support that they require and that there is a clear focus on their full reintegration into society without discrimination.

This last area of focus is particularly important in the context of this paper, allied to the role of European political leadership in delivering fit for purpose, integrated and effective European Beating Cancer and EU Cancer Mission plans which include a focus on cancer survivorship. In general, the overarching attention of the European scientific and clinical communities to date has been more on diagnosis and treatment of cancer than on living with and beyond cancer. However, the fact that nearly 20 million citizens in Europe have survived cancer (https://www.europeancanceracademy.eu/cancer‐survivorship) means that we must greatly enhance our engagement with cancer survivors and the cancer survivorship agenda, in order to ensure that the specific survivorship challenges and needs are adequately addressed, particularly with respect to survivorship research, holistic support for survivors and active rehabilitation and reintegration into society. Survivorship, rehabilitation and reintegration into society are key pillars of the European Cancer Organisation's European Code of Cancer Practice [10]. It is imperative that each cancer patient must have a survivorship care plan [11], while the suggestion for a Cancer Survivor Smart Card or Passport also merits further attention, as exemplified by the Survivorship Passport in paediatric oncology brought forward through continued work in a number of EU projects [12]. A recently published study highlights the importance on capturing detailed European data on cancer cure and quality of life for cancer survivors [13].

In this paper, as members of the Cancer Survivorship Committee of the European Academy of Cancer Sciences, we focus our attention and expertise on identifying the specific survivorship research and innovation challenges that Europe is currently facing and propose a series of cogent recommended solutions that can be adopted by and embedded within the framework of the EU Cancer Mission.

## 4 Enhancing cancer survivorship through research and innovation

Previously, in this journal, we have reviewed the current state of knowledge in‐depth in relation to cancer survivorship and identified specific research areas that should be developed, in order to inform future interventions to support the European cancer survivor and the survivorship agenda in Europe [14]. Additionally, as part of a wider focus on cancer research within Horizon Europe, we highlighted the importance of ensuring that survivorship research is an active component of the EU Cancer Mission [11]. The evidence base from these two papers informs this current publication. Here, we prioritise three distinct Cancer Survivorship Research and Innovation Pillars that we propose should be the areas of focus for a European Cancer Survivorship Research and Innovation Plan. Within these pillars, we delineate a series of challenges that currently limit progress in the cancer survivorship domain (Text Boxes 1, 2, 3) and propose a series of recommended solutions that will firmly embed cancer survivorship research and innovation within the overarching and evolving framework of the EU Cancer Mission.

The three Cancer Survivorship Research and Innovation Pillars that we have identified are as follows:

- The Medical Cancer Survivorship Research and Innovation Pillar
- The Socio‐economic Cancer Survivorship Research and Innovation Pillar
- The Politico‐Legal Cancer Survivorship Research and Innovation Pillar

For each of these Cancer Survivorship Research and Innovation Pillars, we highlight a series of challenges that should be placed firmly within scope of the EU Cancer Mission, accompanied by cogent and relevant solutions that can be successfully implemented across the different upcoming phases of the Horizon Europe research funding programme.

Text Box 1 **Delineating the challenges within the Medical Cancer Survivorship Research and Innovation Pillar**

**Challenges within the Medical Cancer Survivorship Research and Innovation Pillar**

*Challenge 1.1:* Cancer survivorship research is not sufficiently integrated into cancer research activity in Europe.

*Challenge 1.2:* Lack of a European Cancer Survivorship Research and Innovation Plan.

*Challenge 1.3:* Lack of robust data intelligence to underpin cancer survivorship research prioritisation.

*Challenge 1.4:* Research activities often do not underpin cancer survivors' needs.

*Challenge 1.5:* Lack of integration of patients into the research and innovation agenda, with limited active involvement in survivorship research.

*Challenge 1.6:* Limited interdisciplinary research activity in the survivorship domain and a paucity of survivorship research tools.

*Challenge 1.7:* Lack of appreciation of the potential value of the international collaborative research dimension.

*Challenge 1.8:* Paucity of specific research programmes for children, adolescents and young adult survivors.

*Challenge 1.9:* Lack of focus on Palliative/End of Life research.

*Challenge 1.10:* Improve cancer survival such that an average of 70% survival is achieved across Europe by 2035 (the 70:35 vision).

Text Box 2 **Delineating the challenges within the Socio‐Economic Cancer Survivorship Research and Innovation Pillar**

**Challenges within the Socio‐Economic Cancer Survivorship Research and Innovation Pillar**

*Challenge 2.1:* Lack of detailed knowledge of the specific social determinants of cancer inequalities that impact on cancer survivorship.

*Challenge 2.2:* Paucity of relevant tools to assess health‐related quality of life (HRQoL) in cancer survivors.

*Challenge 2.3:* Lack of accurate robust data on the economic burden of cancer for cancer survivors.

*Challenge 2.4:* Paucity of data on the impact and cost‐effectiveness of interventions for cancer survivors.

*Challenge 2.5:* Lack of financial support for cancer survivorship research at European level.

*Challenge 2.6:* Limited integration of social issues into cancer survivorship research activities.

Text Box 3 **Delineating the challenges within the Politico‐Legal Cancer Survivorship Research and Innovation Pillar**

**Challenges within the Politico‐Legal Cancer Survivorship and Innovation Pillar**

*Challenge 3.1:* Limited detailed intelligence on the legal aspects of discrimination for cancer survivors.

*Challenge 3.2:* Lack of research on the legal aspects of reintegration of cancer survivors back into society.

*Challenge 3.3:* Paucity of research that specifically focusses on the activities and requirements of Cancer Patient Advocacy Groups.

*Challenge 3.4:* Lack of knowledge of the stigma associated with cancer.

*Challenge 3.5:* Lack of specific research on survivorship support for patients and for patient empowerment.

### 4.1 Addressing the Medical Cancer Survivorship Research and Innovation Pillar

The challenges posed in the medical cancer survivorship research and innovation domain and the recommended solutions proposed are outlined below:

***Survivorship challenge 1.1:* Cancer survivorship research is not sufficiently integrated into cancer research activity in Europe**.

*Recommended solution 1.1:* Ensure that the cancer survivorship research agenda is an integrated component of the translational cancer research continuum in Europe, so that side effects and late sequelae of treatment can be understood and mitigated against, underpinning a health‐related quality of life (HRQoL) focussed research and innovation agenda. Evaluation of late effects/late complications after cancer treatment ideally should start at the beginning of each clinical trial/cancer treatment intervention by early inclusion of late effect end‐points. Following conclusion of the clinical study, there should be a sustainable system whereby late effects are specifically monitored through long‐term follow‐up, allowing timely capture of new events or complications.

***Survivorship challenge 1.2:* Lack of a European Cancer Survivorship Research and Innovation Plan**.

*Recommended solution 1.2:* Actively support a European Cancer Survivorship Research and Innovation Plan, to be embedded within the EU Cancer Mission, with the ambitious aim of achieving an average of 70% cancer survival in Europe by 2035 (70:35 vision) [15]. The European Society for Paediatric Oncology (SIOPE) manifesto to ‘Cure more, cure better’ (https://worldspanmedia.s3‐eu‐west‐1.amazonaws.com/media/siope/PDF/SIOP‐Manifesto‐A5‐vFINAL.pdf) is highly relevant here and can contribute to this overall vision.

***Survivorship challenge 1.3:* Lack of robust data intelligence to underpin cancer survivorship research prioritisation**.

*Recommended solution 1.3:* Inform prioritisation of cancer survivorship research by performing comprehensive landscape scanning of existing cancer survivorship activities and deploying robust clinical and epidemiologic data to identify, quantify and prioritise specific survivorship research gaps. In this regard, while clinical trial data are highly relevant, survivorship research should also include patients routinely treated within healthcare systems, focussing on clinical effectiveness which should be assessed, ideally, on population‐based patient groups. In order to achieve this and assure effective research activities for all cancer survivors, clinical registries with quality‐assured data are required, combined with long‐term follow‐up to facilitate compilation of data across centres, regions and countries. These registries should collect data on HRQoL. This could form part of the European Health Data Space (https://ec.europa.eu/info/law/better‐regulation/have‐your‐say/initiatives/12663‐A‐European‐Health‐Data‐Space‐), with particular focus on data relating to survivorship. Define different cancer survivor subgroups (including the different ages of survivors, both at treatment and at follow‐up in their survivorship trajectory) on the basis of both clinical and epidemiological data in order to ensure that the survivorship research activity undertaken identifies the most appropriate tailored care and support is delivered to each cancer survivor subgroup as effectively as possible.

***Survivorship challenge 1.4:* Research activities often do not underpin cancer survivors' needs**.

*Recommended solution 1.4:* In prioritising a European Cancer Survivorship Research and Innovation Plan, it is critical to ensure that the chosen priorities clearly reflect survivors' specific long‐term needs (in areas including fertility preservation, reconstruction surgery, mental health, dental health, rehabilitation and others) and that the research is focussed on the cancer survivor community's approved priority areas. Early confirmation of survivors' needs and integration of these needs into the decision‐making process should help inform the key components of, for example, access to rehabilitation (physical, psychological, social, cognitive, nutritional, sexual etc.) and ensure that the European Cancer Survivorship Research and Innovation Plan aligns directly to survivors' needs and wishes. In addition, it is paramount that cancer survivors are active participants in both the design and the implementation of the different components of a European Cancer Survivorship Research and Innovation Plan.

***Survivorship challenge 1.5:* Lack of integration of patients into the research and innovation agenda, with limited patient involvement in survivorship research**.

*Recommended solution 1.5:* As also highlighted in Survivorship Challenge 1.4, ensure that cancer survivorship research and innovation occur in closer collaboration with healthcare professionals, cancer survivors and their patient advocates, mandating that cancer survivors are embedded in the research activities and both provide and receive feedback on the research being performed or proposed. Cocreation of the research and innovation agenda between the cancer survivor community and health professionals is crucial – cancer survivors must be ‘active participants’ rather than ‘passive recipients’ in the process. Additionally, it is important to ensure that all initiatives on survivorship research and innovation should be harmonised in order to avoid fragmentation of efforts and resources.

***Survivorship challenge 1.6:* Limited interdisciplinary research in the survivorship domain and a paucity of survivorship research tools**.

*Recommended solution 1.6:* Promote an interdisciplinary survivor‐centred approach for specific survivorship research programmes. Develop and elaborate new tools to facilitate survivorship research, including the assessment of the health and well‐being of cancer survivors. Bringing together different research communities with added‐value insights provides the best opportunities to deliver optimal solutions that most benefit cancer survivors.

***Survivorship challenge 1.7:* Lack of appreciation of the potential value of the international collaborative research dimension**.

*Recommended solution 1.7:* A comprehensive cancer survivorship research and innovation programme requires international collaboration to help set research priorities, ensure successful implementation and benchmark findings (e.g. ensuring patient‐reported outcomes are captured, reported and compared with data from international partners), so it is important that the European Cancer Survivorship Research and Innovation Plan is both designed and pursued in collaboration with international partners.

***Survivorship challenge 1.8:* Paucity of specific research programmes for children, adolescents and young adult survivors**.

*Recommended solution 1.8:* It is critically important that any European Cancer Survivorship Research and Innovation Plan specifically addresses the needs of children, adolescents and young adults. It is recommended that the EU Cancer Mission includes the ambition to develop age‐adapted research programmes, informed by consultation, that best meet the needs of children, adolescents and young adult survivors. Much work has already been done in this area, particularly by SIOPE and must be built upon.

***Survivorship challenge 1.9:* Lack of Focus on Palliative/End of Life research**.

*Recommended solution 1.9:* Research in palliative and end‐of‐life care is another area of unmet need. This requires a more comprehensive focus on promoting research activities for the full spectrum of palliative cancer patients and at all ages; (a) for those living with a chronic cancer diagnosis under prolonged treatment; (b) for patients in end‐of‐life care; and (c) for patients with very poor prognosis, where patient‐reported outcome measures are challenging.

***Survivorship challenge 1.10:* Improve cancer survival such that an average of 70% survival is achieved across Europe by 2035 (the 70:35 vision)**.

*Recommended solution 1.10:* Support the ambition of a 70:35 vision [14] through a European Cancer Survivorship Research and Innovation Plan that encourages sharing of best practice and promotion of survivorship research and innovation. Long‐term follow‐up of patients should be a target of cancer centres involved in therapy development, in order to implement a survivorship care plan that includes the provision of medical and nonmedical care, while also supporting patient's self‐management, ensuring tertiary prevention and enhancing all aspects of HRQoL. This should include children, adolescents and young adults with certain rare cancers, where little progress has been made in the last 30 years.

### 4.2 Addressing the Socio‐Economic Cancer Survivorship Research and Innovation Pillar

The challenges posed in the socio‐economic cancer survivorship research and innovation domain and the recommended solutions proposed are outlined below:

***Survivorship challenge 2.1:* Lack of detailed knowledge of the specific social determinants of cancer inequalities that impact on cancer survivorship**.

*Recommended solution 2.1:* Promote research to identify the determinants of cancer inequalities linked to social rehabilitation of cancer survivors of all ages, including the disparities present across EU Member States (in particular in Central and Eastern European countries). Studies should be conducted to better assess the specific impact of cancer on people's daily lives, in order to address their needs and target the sources of inequalities that they are experiencing.

***Survivorship challenge 2.2:* Paucity of relevant tools to assess health‐related quality of life (HRQoL) in cancer survivors**.

*Recommended solution 2.2:* Maximise the use of existing tools, including comparisons between different studies and their harmonisation, to ensure best use is made of existing data. Create and evaluate new research tools to accurately assess HRQoL of cancer survivors and to identify the social determinants of health and return to normal life.

***Survivorship challenge 2.3:* Lack of accurate robust data on the economic burden of cancer for cancer survivors**.

*Recommended solution 2.3:* Implement research that performs precise economic evaluations of the indirect costs (including the loss of productivity of the survivor, the social security expenditure, the costs related to the welfare and social security protection of individuals with disabling diseases and the levels of financial toxicity experienced by survivors and their families), in addition to direct health costs associated with survivorship, with the ability to benchmark across European countries and regions. Being able to guarantee an efficient path for cancer patients to recover their health, including supportive care, rehabilitation, palliative and psychosocial care interventions, would guarantee a significant reduction in costs for the social security system, freeing up public resources that could be used to support investment in cancer health innovation. These activities (which should cover all age groups) could align to the cancer inequality dashboard activity proposed at the launch of the Europe Beating Cancer Plan.

***Survivorship challenge 2.4:* Paucity of data on the impact and cost‐effectiveness of interventions for cancer survivors**.

*Recommended solution 2.4:* Evaluate the impact and cost‐effectiveness of supportive care, rehabilitation, palliative and psychosocial care interventions on all age groups of cancer survivors. Outcome research with a focus on cancer survivorship should be a component of the translation cancer research continuum and should be integrated into all National Cancer Control Plans (NCCP) across Europe.

***Survivorship challenge 2.5:* Lack of financial support for cancer survivorship research on a European level**.

*Recommended solution 2.5:* Elaborate proposals that accurately define the financial commitment required to support long‐term cancer survivorship research on a European level (In particular for CEE Countries), so as to ensure that the appropriate level of sustainable budget is dedicated to survivorship research and innovation within the EU Cancer Mission.

***Survivorship challenge 2.6:* Limited integration of social issues into cancer survivorship research activities**.

*Recommended solution 2.6:* Promote social issues such as access to work, education, insurance, loan, mortgage and financial toxicity, to ensure that they are prioritised in the cancer survivorship research and innovation agenda. Research on social issues and factors influencing return to work should be implemented to help safeguard cancer survivors' working lives, their employability, skills and capacity to work. Research on how skills could be offered to self‐employed workers to help them to achieve balance between their health needs and work/social needs should also be encouraged.

### 4.3 Addressing the Politico‐Legal Cancer Survivorship Research and Innovation Pillar

The challenges posed in the politico‐legal cancer survivorship research and innovation domain and the recommended solutions proposed are outlined below:

***Survivorship challenge 3.1:* Limited detailed intelligence on the legal aspects of discrimination for cancer survivors**.

*Recommended solution 3.1:* Promote studies to comprehensively characterise the legal aspects of discrimination for cancer survivors and use this intelligence to inform research on discrimination against cancer patients and how this discrimination can be mitigated.

***Survivorship challenge 3.2:* Lack of research on the legal aspects of reintegration of cancer survivors back into society**.

*Recommended solution 3.2:* Evaluate the adoption of legal provisions that have a key role in promoting reintegration, equality and social inclusion and the Right to Be Forgotten for all age groups [16, 17]. Ensure that the Right to be Forgotten is adopted across all European countries and jurisdictions.

***Survivorship challenge 3.3:* Need for research that specifically focusses on the activities and requirement of Cancer Patient Advocacy Groups**.

*Recommended solution 3.3:* Developing research programmes dedicated to patient advocacy groups (representing childhood, adolescent and adult survivors), including those that identify an appropriate model for financially supporting patient advocacy research activities. This is particularly relevant in the context of the COVID‐19 pandemic, where many Patient Advocacy Groups have experienced significant difficulties in maintaining their activities.

***Survivorship challenge 3.4:* Lack of knowledge of the stigma associated with cancer**.

*Recommended solution 3.4:* Develop appropriate research programmes that define the challenging stigmas associated with cancer, in order to promote a cultural shift to a more active survivorship‐focussed campaign, spreading the key message that cancer is not a death sentence anymore and that once cure has been achieved, cancer survivors have the right to return to a normal life, including the Right to be Forgotten to avoid financial discrimination. Take advantage of the existing legal framework in 4 EU countries (France, Belgium, Luxembourg and the Netherlands) to investigate a pan‐European legal framework on access to financial services, on the basis of equal access for all EU cancer survivors, working closely with existing networks of professional orgaisations, patient advocacy organisations and cancer survivors and their families.

***Survivorship challenge 3.5:* Lack of specific research on survivorship support for patients and for patient empowerment**.

*Recommended solution 3.5:* (A) Determine the potential role of comprehensive survivorship clinics (as exist in the Netherlands for children with cancer) and promote these as examples of good practices to share among EU Countries [18]. Formalise indications on how survivorship care should be organised, whether in specialised survivorship clinics or in rehabilitation clinics or to an entirely different infrastructure that facilitates comprehensive and tailored long‐term follow‐up of individuals.

*Recommended solution 3.5:* (B) Develop and provide supports and tools (including e‐health supports) to empower patients by promoting their self‐management. Online programmes and e‐health tools can help to improve the detection and evaluation of needs in supportive care and offer a robust alternative for educating survivors, since they are considered cost efficient and show equal impact with more conventional approaches. Self‐management programmes need to be offered to cancer survivors that provide advice about potential late effects and their early identification and management.

For both 3.5 (A and B), it will be important to realise that one size does not fit all and we need to be flexible to ensure that local situations in particular countries or regions are taken into account. We need to deliver for all cancer survivors, right across Europe.

## 5 Conclusion

In this policy paper, we build on the previous work of the European Academy of Cancer Sciences that identified a comprehensive evidence base and indicated the absolute need to ensure that cancer survivorship research is recognised as a critical component of the overall cancer research activity in Europe. Here, we articulate the key challenges from a medical, socio‐economic and politico‐legal perspective that need to be recognised and addressed, in order to ensure that cancer survivorship research and innovation are firmly embedded within the European cancer research agenda. We propose a series of recommended solutions to these challenges, which, if acted upon, will contribute to enhance cancer outcomes and improve quality of life for the millions of Europeans who are living with and beyond cancer over the next decade. As part of our recommendations, we call for the establishment of a European Cancer Survivorship Research and Innovation Plan. Integration of this plan within the framework of the EU Cancer Mission would ensure that the challenges that cancer survivors face are clearly delineated, actively researched with sustained funding through future Horizon Europe research funding calls, leading to a greater understanding of the challenges that cancer survivors experience on a daily basis and a research‐informed approach to their resolution.

## Conflict of interest

ML has received honoraria from Pfizer, EMD Serono and Roche for presentations unrelated to this work. ML has received an unrestricted educational grant from Pfizer for research unrelated to this work. FDL, PL, FSM, SN, GS and FM declare no conflict of interest.

## Author contributions

Under the chairpersonship of FM, all authors contributed to a virtual round table and a series of e‐meetings to develop the different ideas that would be presented in the manuscript. All authors contributed to the decisions on the recommendations to be proposed in the manuscript. ML and FM developed the concept of the paper and prepared the first draft. All authors contributed to the final draft of the manuscript.
